# Dual Antibiotic Approach: Synthesis and Antibacterial Activity of Antibiotic–Antimicrobial Peptide Conjugates

**DOI:** 10.3390/antibiotics13080783

**Published:** 2024-08-21

**Authors:** Maria Cristina Bellucci, Carola Romani, Monica Sani, Alessandro Volonterio

**Affiliations:** 1 Department of Food, Environmental, and Nutritional Sciences, Università degli Studi di Milano, Via Celoria 2, 20131 Milano, Italy; cristina.bellucci@unimi.it; 2 Department of Chemistry, Materials, and Chemical Engineering “Giulio Natta”, Politecnico di Milano, Via Mancinelli 7, 20131 Milano, Italy; carola.romani@polimi.it; 3 Consiglio Nazionale delle Ricerche, Istituto di Scienze e Tecnologie Chimica “G. Natta” (SCITEC), Via Mario Bianco 9, 20131 Milano, Italy; monica.sani@polimi.it

**Keywords:** antimicrobial peptides, antibiotics, β-lactams, vancomycin, aminoglycosides, chemical conjugation, combination therapy

## Abstract

In recent years, bacterial resistance to conventional antibiotics has become a major concern in the medical field. The global misuse of antibiotics in clinics, personal use, and agriculture has accelerated this resistance, making infections increasingly difficult to treat and rendering new antibiotics ineffective more quickly. Finding new antibiotics is challenging due to the complexity of bacterial mechanisms, high costs and low financial incentives for the development of new molecular scaffolds, and stringent regulatory requirements. Additionally, innovation has slowed, with many new antibiotics being modifications of existing drugs rather than entirely new classes. Antimicrobial peptides (AMPs) are a valid alternative to small-molecule antibiotics offering several advantages, including broad-spectrum activity and a lower likelihood of inducing resistance due to their multifaceted mechanisms of action. However, AMPs face challenges such as stability issues in physiological conditions, potential toxicity to human cells, high production costs, and difficulties in large-scale manufacturing. A reliable strategy to overcome the drawbacks associated with the use of small-molecule antibiotics and AMPs is combination therapy, namely the simultaneous co-administration of two or more antibiotics or the synthesis of covalently linked conjugates. This review aims to provide a comprehensive overview of the literature on the development of antibiotic–AMP conjugates, with a particular emphasis on critically analyzing the design and synthetic strategies employed in their creation. In addition to the synthesis, the review will also explore the reported antibacterial activity of these conjugates and, where available, examine any data concerning their cytotoxicity.

## 1. Introduction

Antibiotics are a class of drugs, being among the highest prescribed drugs worldwide, specifically designed to combat bacterial infections by either killing bacteria or inhibiting their growth [1]. Discovered in the early 20th century, antibiotics revolutionized medicine and have saved countless lives by effectively treating previously lethal infections [2,3]. These medications work through various mechanisms, such as inhibiting or disrupting cell wall synthesis, protein production, DNA replication in bacteria, or interfering with metabolic pathways [4,5]. Despite their critical role in healthcare, the overuse and misuse of antibiotics have led to a growing issue of antibiotic resistance, where bacteria evolve mechanisms to evade the effects of these drugs [6,7,8]. Moreover, the fact that most antibacterial agents in use today are derived from natural products synthesized by bacteria or fungi to defend against bacterial competitors further facilitated the development of antimicrobial resistance (AMR). This resistance poses a significant challenge to global health, necessitating ongoing research and the development of new antibiotics and alternative treatments which are not advancing as fast as resistance [9]. Indeed, it is projected that by 2050, the annual fatalities could exceed 10 million unless appropriate measures are implemented, and research and development efforts are intensified to address AMR [10]. 

The most urgent need for new antibiotics is dictated to combat resistant strains, especially Gram-negative bacteria *P. aeruginosa* and *E. coli*, which have a second polar outer membrane and numerous efflux pumps that make them less susceptible to drug intervention, defined as critical pathogens, and to a slightly lesser extent, methicillin-resistant *S. aureus* (MRSA), defined as high priority [11]. Despite extensive screening efforts with traditional drug-like libraries, very few non-natural product-derived antibacterial agents have been discovered [12,13]. It has become increasingly apparent that the physicochemical properties required to evade these bacterial defenses differ from those needed for traditional drugs since antibiotics are generally more polar and larger than drugs targeting other conditions [14]. Having evolved over millennia to target bacteria, antibiotic natural products inherently possess the necessary attributes to overcome bacterial defenses and have thus been successfully used for decades as starting points for semi-synthetically derived next-generation antibiotics [15]. However, discovering new antibiotics is challenging for several reasons. Firstly, it is extremely difficult to identify new scaffolds that are both effective and safe [12,16]. Additionally, bacteria can quickly develop resistance to new antibiotics through mutations and horizontal gene transfer, often making new drugs ineffective shortly after their introduction [6]. Furthermore, economic and regulatory barriers also pose significant obstacles to the development of new antibiotics. Developing a new antibiotic is expensive, often costing upwards of a billion dollars, which includes costs for discovery, preclinical testing, clinical trials, and regulatory approval [17]. Furthermore, antibiotics are typically used for short durations, unlike treatments for chronic diseases, resulting in lower financial returns and the pharmaceutical market prioritizes drugs with higher profitability, such as those for chronic conditions or lifestyle diseases, leading to underinvestment in antibiotics [18]. To compound the issue, new antibiotics are often held in reserve to delay resistance, further limiting sales. Moreover, regulatory agencies require extensive testing to ensure safety and efficacy, which can be particularly challenging for antibiotics due to the need for novel approaches and considerations of resistance [19]. Additionally, continuous post-approval surveillance for resistance and effectiveness in real-world use is necessary, adding to the complexity and cost of antibiotic development [20]. Nonetheless, some new antibiotics have been launched in the market during the last twenty years, demonstrating that the quest to fight AMR with small molecules is not at a dead end [21]. 

One strategy to avoid the challenging search for new scaffolds [22] and to chase an old antibiotics renaissance is to administer two or more antibiotics simultaneously to obtain a synergistic effect in the so-called combination therapy [23]. This approach can enhance the efficacy of treatment by attacking pathogens through different mechanisms, reducing the likelihood of resistance development. By combining drugs with complementary actions, combination therapy can achieve synergistic effects, lower required dosages, and minimize toxicity. Additionally, it can target a broader spectrum of pathogens, including multi-drug-resistant strains [24]. However, the strategy may not always work in vivo because of the different pharmacokinetics of the antibiotics [25]. Differences in the absorption, distribution, metabolism, and excretion of the drugs can lead to suboptimal levels of one or more components, reducing overall efficacy and increasing the risk of side effects. Moreover, there is also a risk of synergistic toxicity, where the combined drugs produce greater adverse effects than expected. This can limit the dosage and effectiveness of the treatment.

Alternatively, combination therapy involving conjugates is an innovative approach to fighting AMR. This strategy involves linking an antimicrobial agent with another molecule, such as a drug, peptide, or targeting ligand, to enhance its effectiveness and specificity [26,27]. Conjugates have shown promise in preclinical studies and are being explored for their potential to target a wide range of pathogens, including multi-drug-resistant bacteria [28]. Particularly interesting are the conjugates between conventional antibiotics and antimicrobial peptides (AMPs) because they possess several advantages compared to conjugates between small-molecule antibiotics. Apart from enhanced activity due to a synergistic effect and/or broader spectrum of activity, AMPs can significantly enhance the effectiveness of antibiotics through improved penetration and targeted delivery. Indeed, AMPs facilitate the penetration of antibiotics into bacterial cells by interacting, disrupting, or translocating cell membranes, thereby increasing the intracellular concentration of the antibiotic [29]. Furthermore, conjugation can enhance the specificity of antibiotics towards bacterial cells over host cells, reducing off-target effects and toxicity [30]. Moreover, the enhanced potency of antibiotic–AMP conjugates allows for lower dosages, reducing potential side effects and toxicity [31]. Improved targeting and reduced doses also minimize the risk of adverse effects, leading to safer treatments for patients [32]. Lastly, the conjugates can be designed to incorporate a variety of AMPs and antibiotics with suitable linkers, allowing for tailored treatments against specific pathogens and resistance profiles.

This review aims to describe the rationale behind the design of the antibiotic–AMP conjugates that appear in the literature [33], encompassing de facto AMPs and cell-penetrating peptides (CPPs), the strategies used for their synthesis, and briefly discuss their activity against the bacterial targets. This review is not intended to give a full overview of the different classes of antibiotics, AMPs, and their mechanisms of action, which will be only summarized in the following two sections for the sake of a better comprehension of the main topic. Readers interested in a more detailed discussion on the features of the single class of antibiotics or AMPs can refer to the reviews cited therein.

## 2. Small-Molecule Antibiotics

**Figure 1:**
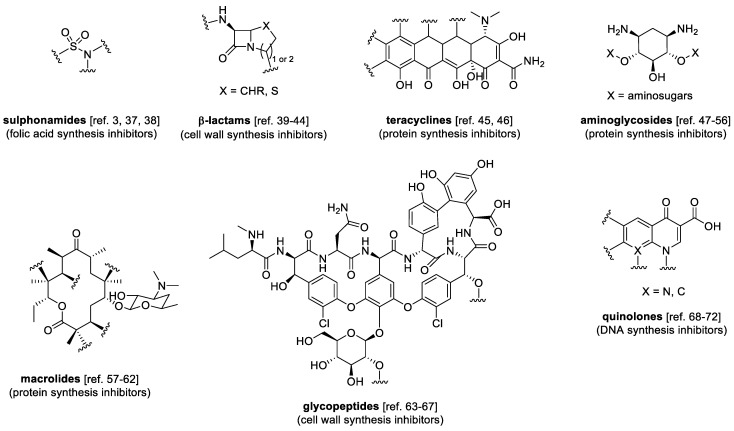
General structure of the main classes of antibiotics [3,37,38,39,40,41,42,43,44,45,46,47,48,49,50,51,52,53,54,55,56,57,58,59,60,61,62,63,64,65,66,67,68,69,70,71,72].

Small-molecule antibiotics, both unconsciously and consciously, have been used in various forms for thousands of years [2]. The identification of salvarsan as an anti-syphilis drug in 1909 [34], followed by the serendipitous discovery of broad-spectrum antimicrobial penicillin by Fleming in 1928 [35], opened an incredibly prolific era during which different small molecule antimicrobials were developed and marketed. More specifically, we witnessed two decades referred to as “the golden age of discovery” of antibiotics, during which different classes of natural antibiotics were isolated from natural sources and identified, followed by a second “golden age of medicinal chemistry”, where successive generations of the natural scaffolds were developed by chemical modification [36]. Both natural and synthetic antimicrobials can be classified according to the chemical core structure and mechanism of action (Figure 1). 

Sulfonamides are an important class of antibiotic drugs with a broad spectrum of activity, highly effective against gram-positive bacteria and some gram-negative bacteria, which were serendipitously discovered in the chemical dyes industry [3]. Sulfonamides act as competitive antagonists and structural analogs of p-aminobenzoic acid (PABA) in the synthesis of folic acid, which is essential for bacterial DNA production [37]. Due to chemical modification, they have witnessed 80 years of continuous use, not only as antibiotics, but also as diuretics, antidiabetics, antiarrhythmics, and COX2 inhibitors [38]. With the advent of penicillin and increasing antibiotic resistance, the demand of new sulfonamides witnessed a decrease. Indeed, after the discovery of penicillin, β-lactam antibiotics encompassing penicillin and following generation cephalosporins, carbapenems, penems, and monobactams (Figure 1) became probably the most popular class of antibiotics, revolutionizing the treatment of infectious diseases [39]. Their action depends on the structure of the constrained β-lactam four-membered ring common to all the generations, which renders the amide bond prone to react with alcohols, and in particular, with the side chain of serines belonging to the so-called penicillin-binding peptides responsible for the cell wall synthesis [40,41]. However, over the past 60 years, resistance to penicillins—mostly due to the microbial production of β-lactamases, but also to conformational changes in penicillin-binding proteins, permeability changes in the outer membrane, and activation of efflux pumps—has been steadily increasing so much that there are serious concerns that β-lactams may soon become ineffective against deadly bacterial infections [42,43,44].

Discovered in the 1940s, tetracyclines are a family of antibiotics that inhibit protein synthesis by preventing the attachment of aminoacyl-tRNA to the ribosomal acceptor (A) site [45]. Tetracyclines are broad-spectrum agents, effective against a wide range of gram-positive and gram-negative bacteria. Their favorable antimicrobial properties and lack of major adverse side effects have led to extensive use in treating infections in both humans and animals. However, during the two decades of the 1970s and 1980s, tetracyclines became a second choice for treatment because of the development of the growing availability of other antibiotics and acquired resistance mainly due to two major mechanisms such as efflux pumps and ribosomal protection proteins. Chemical modification, mostly performed on the D cycle of the scaffold, led to the development of three different generations of tetracyclines, the latest encompassing eravacycline, omadacycline, and tigecycline, which were approved within the past 15 years, representing a new era in the use of these antibiotics (Figure 1) [46].

Also, the era of aminoglycoside antibiotics started in the 1940s when streptomycin was discovered [47]. Aminoglycosides are a class of natural and semi-synthetic polyamino sugars, most of them having in common a central cyclohexane ring referred to as 2-deoxystraptamine (2-DOS), to which different aminosugars are bonded through glycosidic linkage (Figure 1) [48]. Aminoglycosides have been widely applied against many types of Gram-positive and Gram-negative pathogens, which are still being widely used worldwide due to their ability to interact mainly through electrostatic interaction but also through hydrogen bond networking with the A-site of the 16S ribosomal RNA and to the 50S ribosomal subunit, perturbing the “proof-reading” process that ensures protein translation, and inhibiting translocation and ribosome recycling, respectively [49,50,51]. Throughout time, pathogens have developed three different mechanisms of bacterial resistance. The principal mechanism involves the inactivation of aminoglycosides by a family of enzymes referred to as aminoglycoside-modifying enzymes (AMEs), expressed by resistant strains that are able to acetylate the amino groups or phosphorylate the hydroxy functions. Resistance can also arise through mutations in the ribosomal target or, increasingly, through the modification of the ribosome by ribosomal methyltransferase enzymes. Lastly, the alteration of the bacterial cell wall by acquired lipid modifications that repel highly polar aminoglycosides improves impermeability, and the activation of efflux pumps results in a lower concentration of aminoglycosides in the bacterial cells [52]. To overcome and fight antibacterial resistance, many derivatives of aminoglycosides have been synthetized, starting from the natural scaffolds [53,54]. Amphiphilic aminoglycosides, aminoglycoside heteroconjugates, aminoglycoside homo- and heterodimers, as well as conformationally restricted aminoglycosides, have been synthetized and tested, providing a better knowledge of their mechanism of action and paving the way for new applications of aminoglycosides as antifungal, anticancer, and gene/drug delivery vectors [55,56].

Macrolide antibiotics, renowned for their safety and high efficacy against Gram-positive bacteria, have been extensively used in clinical settings for over 50 years since the first antibiotic, pikromycin, was isolated in 1950 [57]. Natural macrolide antibiotics share a common structural feature, i.e., a macrocyclic lactone of different sizes (from 12 to 15 members) bearing one or more amino- or deoxy-sugars (Figure 1), erythromycin being the most popular [58]. As members of the largest class of antibiotics, they are particularly effective in treating upper and lower respiratory tract infections by reversible binding to the 23S rRNA at or near the peptidyl transferase center, thus inhibiting the synthesis of bacterial proteins [59]. However, despite their excellent antibacterial activity, macrolides often suffer from poor bioavailability, unpredictable pharmacokinetics, and low stability in the acidic environment of the stomach. These limitations, along with the emerging antibacterial resistance, prompted early efforts to develop new derivatives with enhanced properties, raising a growing interest in the synthesis of the next generations of macrolide antibiotics. The second generation of macrolide antibiotics are semisynthetic derivatives, mostly derived from erythromycin [60,61], whereas a third generation has been prepared through a fully synthetic platform technology by assembling a left-hand component and a right-hand component through reductive amination and subsequent macrolactonization [62].

During the same period, the first glycopeptide vancomycin was isolated and readily commercialized by Eli Lilly in 1958, paving the way to the glycopeptide antibiotics era to fight *Staphylococcus aureus* (*S. aureus*), a microorganism able to elude most of the classic antibiotics [63]. Glycopeptide antibiotics are a class of heptapeptides that are sub-classified according to the identity of amino acids in positions 1 and 2 (Figure 1) [64]. Biochemical studies indicate that vancomycin and other glycopeptides inhibit peptidoglycan synthesis by forming a stoichiometric 1:1 complex with the peptidoglycan precursor UDP-N-acetylmuramylpentapeptide through five hydrogen bonds with the acyl-D-ala-D-ala moiety, eventually inhibiting the transglycosylase enzyme and transpeptidase enzyme reactions fundamental to the synthesis of the rigid cell wall peptidoglycan [65]. Prior to 1984, the glycopeptide class included only a few members beyond vancomycin, teicoplanin, ristocetin, and avoparcin. However, due to administration issues and side effects, and with the acknowledgment of the threat posed by antibiotic resistance, the class of glycopeptides swelled to include thousands of natural and semi-synthetic compounds possessing multiple mechanisms of action. The semisynthetic approaches to designing glycopeptide second generation can be broadly categorized into three strategies, namely (1) the functionalization of the functional groups on the outer shell of the parent peptides, (2) the modification of the amino acids through disassembling and reassembling the peptidic scaffold, and (3) dimerizing or trimerizing the glycopeptide through covalent bonding [64,66], the first one probably being the most exploited [67].

At the end of the “golden age of discovery” of antibiotics, the first quinolone, nalidixic acid, was isolated and suddenly introduced into the clinic [68]. However, quinolones became the most often prescribed antibiotics in the world to treat a range of microbial diseases in humans only in the 1980s, when the second generation of this class of antibiotics was synthetized [69]. The common structural features of such compounds are a bicyclic skeleton, a carboxylic acid, and a keto group at positions 3 and 4, respectively, which are necessary for their pharmacological activity (Figure 1) [70]. Quinolones act as DNA synthesis inhibitors because they are able to bind bacterial enzymes DNA gyrase and DNA topoisomerase IV, which are enzymes that play fundamental roles in most nucleic acid processes, and convert them into cellular toxins [71]. Like the other class of antibiotics, the targets of quinolones have also developed antibiotic resistance, mainly through three different mechanism: (1) target-mediated resistance due to the modification of gyrase and topoisomerase IV structures, (2) plasmid-mediated resistance due to the presence of enzymes able to acetylate the free nitrogen on the quinolone scaffold and the generation of efflux pumps, and (3) chromosome-mediated resistance though the underexpression of porins and overexpression of efflux pumps, leading to a decrease in concentration of the drugs in the cell. Due to the relative simplicity of the scaffold and to their wide-spectrum anti-infective efficacy, quinolones have received a lot of interest from the medicinal chemistry field in the search of novel derivatives [72].

## 3. Antimicrobial Peptides (AMPs)

Since the isolation of gramicidin A and B in the 1930s, AMPs have emerged as promising candidates against antimicrobial resistance due to their broad-spectrum activity and multiple/unique modes of action [73,74,75]. AMPs, also known as host defense peptides, are a class of naturally occurring molecules that play a crucial role in the innate immune systems of animals, plants, insects, and microorganisms [76]. They show activity against Gram-positive and Gram-negative bacteria and other pathogens, such as fungi, viruses, and parasites [77,78]. Moreover, they can also exert potent antibiofilm activity against multiresistant bacteria [79]. Currently, more than 60 peptides have been approved by the FDA and more than 400 are in clinical trials, but only 7 have reached the market, used mainly as topical medications and, in cases involving serious infections, as injectables [80,81]. 

Structurally, AMPs are small peptides, from 12 to 50 amino acids in length, and typically share several common physicochemical features, namely amphipathicity and the capacity to form stable secondary motifs (α-helical, β-sheet, mixed, and cyclic structures) which are responsible for their biological activity [82]. Amphipathicity contributes significantly to the AMPs’ ability to selectively target microbial membranes. In fact, their hydrophilic region, primarily composed of cationic residues (Lys and Arg), plays a fundamental role in the initial binding to negatively charged components on bacterial membranes through electrostatic interactions. Unlike mammalian cell membranes, bacterial membranes are rich in negatively charged components, such as phospholipids in the peptidoglycan cell wall of Gram-positive bacteria, and lipopolysaccharide in the outer membrane of Gram-negative bacteria. Once bound, AMPs exert their antimicrobial effects mainly through two different mechanisms: bactericidal (membrane disruption causing cell lysis) and bacteriostatic (metabolic processes interference through nucleic acids binding and modulation of bacteria essential functions) [83,84]. 

The membrane-targeting mechanisms of AMPs can be described through three different models: the barrel stave, the toroidal pore, and the carpet. In the barrel-stave model, AMPs are inserted into the lipid bilayer of the microbial membrane, aligning themselves perpendicularly to the plane of the membrane. The peptides aggregate to form a pore or channel through the membrane, resembling the staves of a barrel. The formation of these transmembrane channels leads to the uncontrolled leakage of ions and other small molecules, disrupting cellular homeostasis and ultimately causing cell death [85]. Similar to the barrel-stave model, AMPs in the toroidal pore model are inserted into the membrane. However, unlike barrel-stave pores, toroidal pores cause the lipid monolayers to bend continuously through the pore, resulting in a toroidal (doughnut-like) structure. This bending disrupts the integrity of the membrane, allowing ions and other molecules to pass through the pore, leading to cell death [86]. In the carpet model, AMPs align parallel to the membrane surface, covering it like a carpet. The peptides interact with the lipid head groups, leading to a destabilization of the membrane. As the concentration of peptides increases, this destabilization causes the membrane to disintegrate in a detergent-like manner, resulting in cell lysis.

Initially, the bactericidal effects of AMPs were attributed only to membrane-active mechanisms. However, it has now been recognized that many AMPs target essential cellular components and functions, leading to bacterial death. These AMPs translocate into the cell membrane without disturbing it and inhibit critical cellular processes by interacting with intracellular targets. To date, several mechanisms have been identified, including the inhibition of protein and nucleic acid synthesis, as well as the degradation of enzymes and proteins [87]. Notably, some AMPs exhibit multiple modes of action, killing bacteria by both disrupting their membranes and interacting with intracellular targets [88]. One well-known intracellular target is genomic DNA. AMPs can bind to DNA, affecting the expression of related genes and inhibiting the synthesis of essential macromolecules. This interaction can also lead to the degradation of DNA, further hampering bacterial survival [89]. The ribosome, a key component of the translation machinery, is another significant target for AMPs. By interacting with 70S ribosomes, AMPs can inhibit protein synthesis. This interaction is facilitated by multiple hydrogen bonds and stacking interactions, effectively halting bacterial growth and replication [90]. Intracellular enzymes and organelles can also be targets for AMPs. By inhibiting or degrading essential enzymes, AMPs disrupt vital metabolic processes within bacterial cells. This inhibition can result in the accumulation of toxic intermediates or the depletion of necessary substrates, leading to cell death [91]. 

## 4. Antibiotic–AMP Conjugates

The synergistic effects of antimicrobial peptides with conventional antibiotics represent a promising strategy to enhance antibacterial efficacy and combat antibiotic resistance. In fact, by combining different mechanisms of action, this approach can overcome bacterial defenses and reduce the likelihood of resistance development [92]. One primary mechanism by which AMPs enhance the efficacy of conventional antibiotics is through membrane disruption. Many AMPs destabilize bacterial membranes, increasing permeability and allowing antibiotics to penetrate more easily. This increased uptake can significantly enhance the antibacterial activity of antibiotics that typically have limited access to intracellular targets. This synergistic effect, which can be obtained by physical coadministration of the antibiotics or by the synthesis of covalently linked conjugates, is particularly relevant for antibiotics which require access to the bacterial inner compartments to exert their effects [93,94]. 

Antibiotic–AMP conjugates are synthetized by anchoring conventional antibiotics to an AMP or CPP through a suitable bifunctional linker. The peptide has two points of attachment, the N-terminus or the C-terminus, even if the selective use of a side chain could, in theory, be taken into consideration. In general, the linker can be classified as a stable covalent linker or a cleavable stimuli-responsive linker. With cleavable stimuli-responsive linkers, the AMP and the antibiotic could each act independently upon entering the bacterial cell, targeting their respective sites. Conversely, if the conjugate molecules remain intact, they function as a single, multimodal antibacterial compound. This allows them to bind to and affect their targets simultaneously, with dynamics that may differ from those of the individual components. Probably the most problematic task for the synthesis of these conjugates is to find the right point of attachment on the antibiotic, since they usually possess many reactive functional groups in their scaffolds. Accordingly, the following sections of this chapter are organized considering the antibiotic scaffold.

### 4.1. Vanconomycin–AMP Conjugates

**Figure 2:**
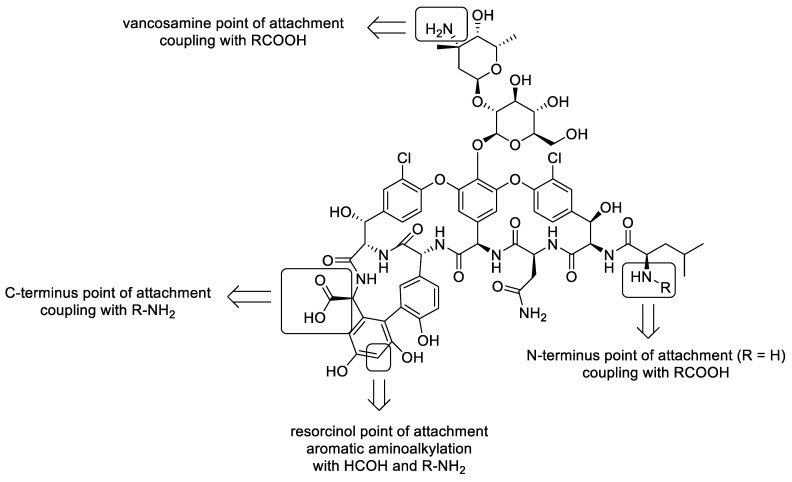
Vancomycin’s points of attachment.

Vancomycin, exhibiting one of the strongest bindings known for low-molecular-weight organic compounds with the D-Ala-D-Ala motif of the cell wall precursor lipid II, was initially considered the drug devoted to treating antibiotic-resistant bacteria, as it is immune to the development of resistance [64,65,66]. However, 30 years after its discovery, different vancomycin-resistant strains, such as *Enterococcus faecium* (VRE), vancomycin-intermediate and -resistant *Staphylococcus aureus* (VISA and VRSA) have been observed, for which the discovery and development of novel antibiotics are urgently needed. In this contest, since clinical resistance to vancomycin took a lot of time to arise, the modification of this glycopeptide could be a successful strategy. From a synthetic chemical perspective, the selective functionalization of vancomycin could seem very difficult due to the presence of many functional groups. However, it has been shown that there are four functional groups, referred to as points of attachment, which can be exploited for selective functionalization due to their unicity or particular reactivity (Figure 2). First, the carboxylic acid at the C-terminus of the peptide sequence is the only carboxy functional group present in the vancomycin scaffold, and thus can selectively react with amines upon worthy activation. Also, the amino functional group at the N-terminus can be selectively coupled with activated carboxylic acids, but only when it is not methylated (R = H, norvancomycin). Indeed, in vancomycin (R = Me), activated carboxylic acids react with the amino group in the glycosyl moiety (vancosamine, third point of attachment). Finally, the fourth point of attachment is the resorcinol aromatic carbon in the ortho position of the two hydroxy groups that, being very electron rich, readily undergo electrophilic aromatic substitution with iminium salts in situ, produced by the reaction of formaldehyde and primary amines.

**Figure 3:**
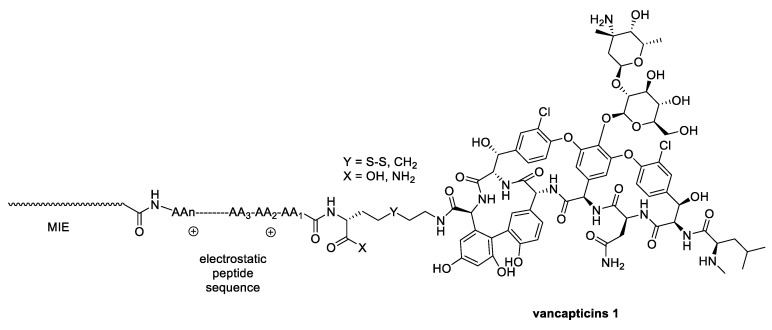
Structure of vancapticins **1**.

One way to face vancomycin-resistant strains is to modulate the structure of the drug to increase membrane binding and selectivity, eventually enhancing drug concentration at the target site [95]. Accordingly, a library of vancomycin derivatives, referred to as vancapticins **1**, was designed by coupling the free carboxylic acid on the glycopeptide with different cationic peptides, mostly polilysines, having distinct lipophilic membrane-insertive elements (MIEs) tethered at the N-terminus through two linkers, one of them built on a cleavable disulfide bond (Figure 3). Structure–activity relationship studies (SAR) revealed that vancapticins **1** possess enhanced membrane affinity, which boosts their effectiveness against MRSA and various other Gram-positive bacteria. Additionally, vancapticins **1** retain their potency against strains that are resistant to traditional glycopeptides.

**Scheme 1:**
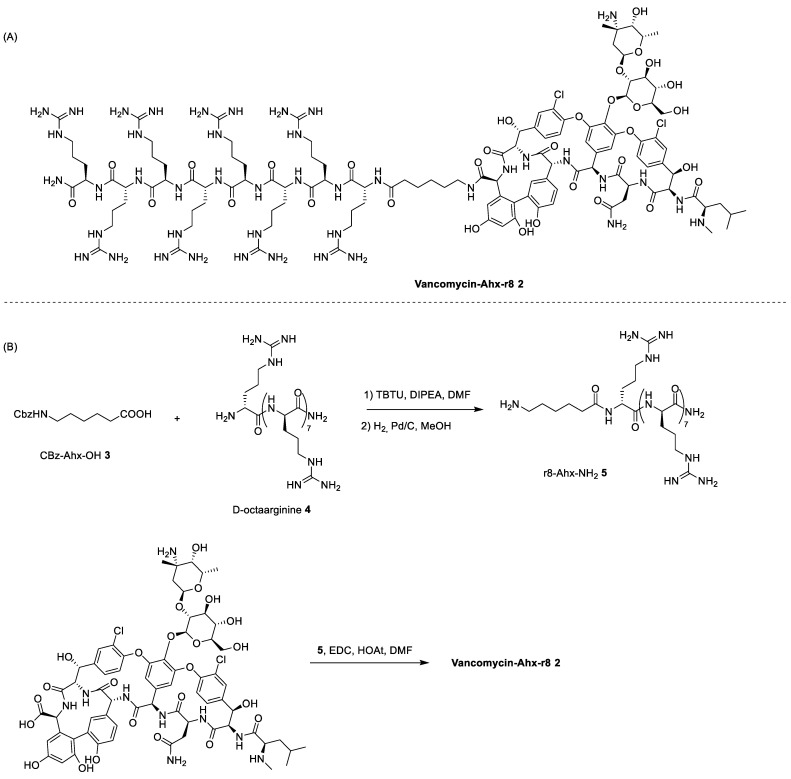
Structure of vancomycin-Ahx-r8 conjugate **2** (**A**) and its synthesis (**B**).

The same strategy, namely tethering polycationic peptides to vancomycin to fight antibacterial resistance, have been exploited for the synthesis of two vancomycin-polyarginine conjugates [96]. Exploiting again the reactivity of the free carboxylic acid, vancomycin was tethered to the N-terminus of D-octaarginine (r8) through a non-cleavable aminohexanoic acid (Ahx) linker, obtaining conjugate **2** (Scheme 1A), which could have a stronger affinity for the surface of the cell membrane and enhanced cell permeability, facilitating the action of vancomycin in arresting the cell wall synthesis and giving to vancomycin access to intracellular binding targets. Octaarginine R8 **4** was prepared through solid-phase peptide synthesis (SPPS) and coupled with Cbz-Ahx-OH **3** in solution, leading to the formation of r8-Ahx-r8 **5** after the hydrogenolysis of the Cbz protecting group (Scheme 1B). Intermediate **5** was finally coupled to vancomycin, producing the final conjugate vacomycin-Ahx-r8 **2**, which turned out to be much more active than vancomycin by orders of magnitude against difficult-to-treat MRSA populations, such as biofilms and persister cells, maintaining comparable minimal inhibitory concentration (MIC) against vancomycin-resistant Gram-positive organisms such as VISA and VRE.

**Scheme 2:**
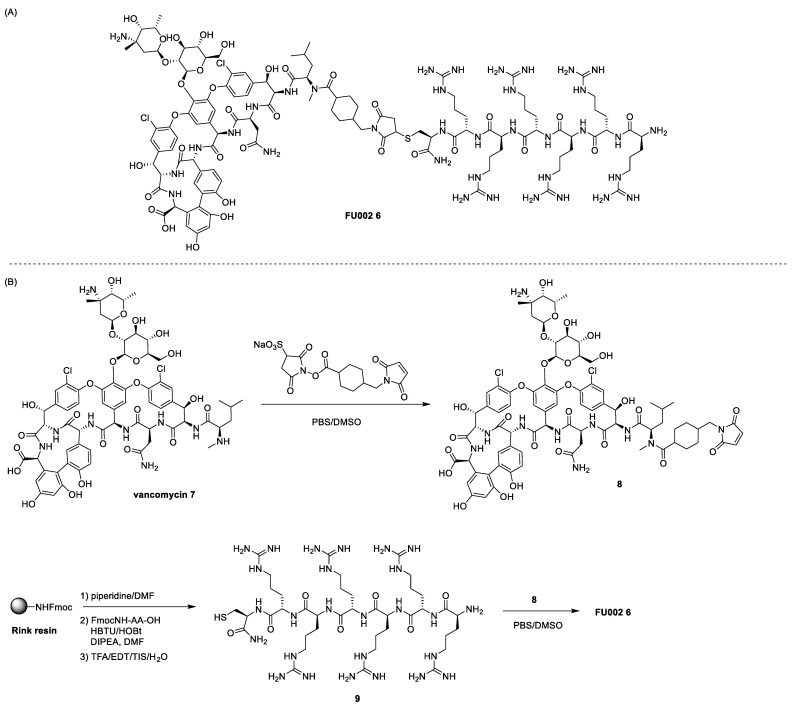
Structure of FU002 conjugate **6** (**A**) and its synthesis (**B**).

Following the same rationale, different vancomycin-polyarginine conjugates were synthetized at four distinct points of attachment, namely the free carboxylic acid (V_C_), the carbon in ortho position to the hydroxy groups of the resorcinarene ring (V_R_), the N-methylammino function on the leucine residue (V_N_), and the free amino function on the glycosyl frame (V_V_) (in Scheme 2A the structure of the most performant V_N_ derivative FU002 **6** is represented) [97]. Apart from the site of attachment, the structures of the conjugates are very similar, composed of the vancomycin antibiotic **7**, a heterobifunctional cross-linker, and hexaarginine, tagged with Cys at the C-terminus **9**. The lead candidate FU002 **6** was prepared by site-specific coupling of vancomycin with sulfo-succinimidyl 4-(N-maleimidomethyl)cyclohaxane-1-carboxylate (sulfo-SMCC), providing intermediate **8**, which was clicked in a solution with Cys-(Arg)_6_-NH_2_ **9**, affording FU002 **6** (Scheme 2B). FU002 **6** showed a remarkably increased activity against the most important types of vancomycin-resistant bacteria, having an additional mechanism of action beyond the interaction with the D-Ala-D-Ala moiety responsible for cell-wall synthesis and superior pharmacokinetics.

**Figure 4:**
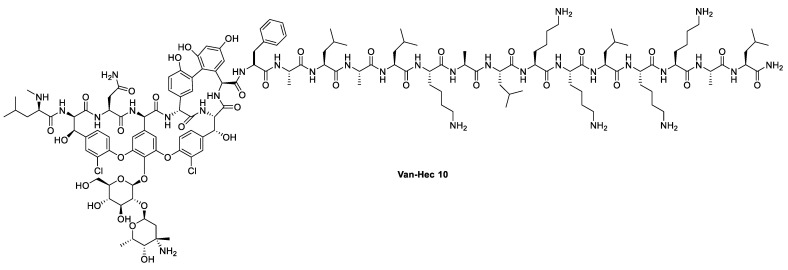
Structure of Van-Hec **10**.

With the aim to increase the cell-permeability of vancomycin but also to exploit the synergistic effect of two antibiotics belonging to different classes, Adams et al. conjugated vancomycin to amphiphilic AMPs Hectate (Hec), which is an amphiphilic peptide with a net-positive charge and α-helix-predominant conformation (Figure 4) [98]. Exploiting the reactivity of the free carboxylic acid on vancomycin and without the use of any linker, vancomycin and Hec were coupled in a solution producing a Van-Hec conjugate **10** [99]. The synergistic effect of vancomycin conjugate to Hec, different from the two antibiotics alone, causes the disruption of the bacterial cell-wall integrity, and is thus very active against wild-type MRSA and VRSA.

**Scheme 3:**
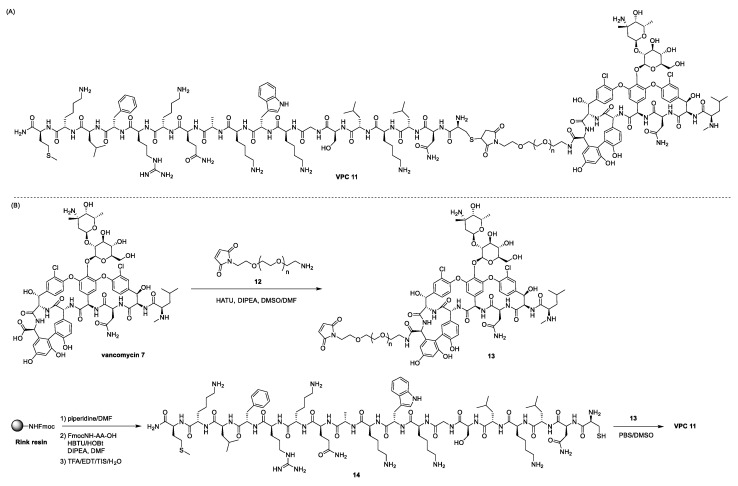
Structure of VPC conjugate **11** (**A**) and its synthesis (**B**).

Inspired by the vancomycin conjugates shown above and considering that Gram-negative strains are challenging to fight due to the presence of the impermeable lipopolysaccharide (LPS)-rich outer membrane [100], a novel series of conjugates was recently designed and synthetized by tethering vancomycin to antimicrobial LPS-binding peptides that were previously demonstrated to exhibit a strong effect against Gram-negative bacteria [101]. Actually, a library of 80 conjugates, referred to as vancomycin-LPS-binding peptide conjugates (VPCs) has been synthetized, exploiting all four points of attachment on the vancomycin scaffold, different chemical inert bifunctional linkers, comprising alkyl and PEG linkers, and a collection of six LPS-binding peptides [102]. After a first generation of conjugates, where short peptides were tethered through click azide-alkyne reaction, which showed modest MICs against Gram-negative strains—even if better activity than vancomycin against *E. faecium* Gram-positive strain—a second generation of VPCs was prepared, of which the structure of the most active, conjugate VPC **11**, is shown in Scheme 3A. For this second generation of conjugates, a different chemical strategy was chosen, namely the functionalization of the vancomycin core **7** with bifunctional linkers **12**, leading to the formation of intermediate **13**, having a maleimide moiety at the other end of the vancomycin point of attachment, which clicked in solution with Cys-functionalized LPS-binding peptides **14**, previously prepared in solid phase (SP) (Scheme 3B). Some of these conjugates were further functionalized on a different vancomycin point of attachment with lipophilic tails to study the synergistic effect of the latter and LPS-binding peptides when tethered to the vancomycin scaffold. The results in terms of MICs of VPNs compared to vancomycin against Gram-positive and Gram-negative strains showed an increase in activity against VRE and Gram-negative strains such as AB1157 (*E. coli*), *A. baumannii,* PA01 (*P. aeruginosa*), and *K. pneumonia*, showing that modification with LPS-binding peptides (and further lipophilic tails) alters the antimicrobial profile of vancomycin when fighting Gram-negative bacteria.

### 4.2. β-Lactams-AMP Conjugates

**Figure 5:**
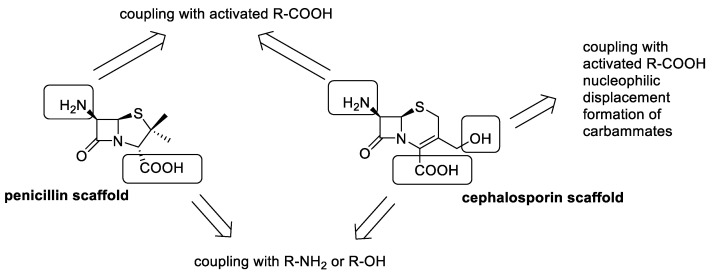
β-lactam’s points of attachment.

With the evolution of many microorganisms that developed resistance to β-lactam antibiotics, mainly due to the widespread diffusion of β-lactamase enzymes, a huge effort has been and is being devoted by the scientific community in the quest for new derivatives that will eventually lead to new generations of these antibiotics. Since the β-lactam ring, the main feature for their activity is very labile, and the vast majority of the β-lactam analogs arose from chemical modification of the pharmacophoric scaffold rather than total synthesis [39]. Actually, both the penicillin scaffold and cephalosporin scaffold possess two functional groups, namely the amino and the carboxylic acid groups, easily derivatized upon protection of the other (Figure 5). Moreover, cephalosporin possesses a further point of attachment consisting of the hydroxy group at the 3′ position.

**Scheme 4:**
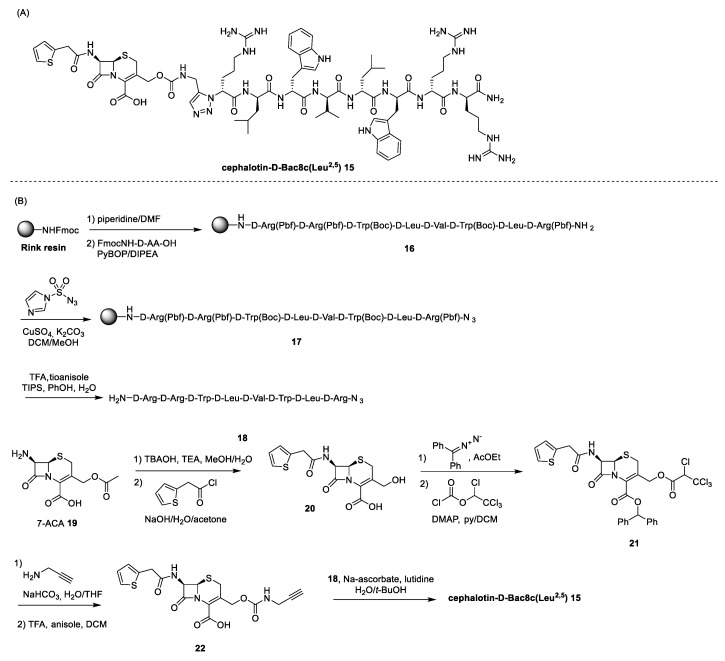
Structure of cephalotin- D-Bac8c(Leu2,5) conjugate **15** (**A**) and its synthesis (**B**).

One of the drawbacks that limits the use of AMPs as therapeutic agents is their toxicity mainly caused by the presence of different cationic moieties. Indeed, it has been shown that after blocking the amino groups of polymyxin E as methane sulphonate, the resulting prodrug can be used systematically [103]. Inspired by the prodrug concept, the first β-lactam-AMP conjugate was synthetized by linking cephalotin to D-Bac8c(Leu2,5), an enantiomeric derivative of AMP Bac8c where the two D-isoleucine amino acids in positions 2 and 5 are substituted with D-leucine, producing a conjugate, cephalotin- D-Bac8c(Leu2,5) **15**, which has reduced net-positive charge due to the presence of a carboxylate (Scheme 4A) [104]. The point of attachment chosen was the hydroxy group of commercially available 7-aminocephalosporanic acid (7-ACA). Accordingly, 7-ACA **19** was first deacetylated to obtain the required free 3′-OH group by using mild tetrabutylammonium hydroxide (TBAOH) at low temperature to avoid the β-lactam amide hydrolysis, then reacted with thienylacetyl chloride, producing the amide intermediate **20** (Scheme 4B). Next, after protection of the carboxylic acid as diphenylmethyl ester, the OH group was converted into the corresponding tetrachloroethyl carbamate **21**, which was finally transformed in **22** by reaction with propargyl amine, followed by the ester protecting group cleavage in acidic conditions. Resin-bound D-Bac8c(Leu2,5)-NH_2_ **16** was prepared by standard SPPS according to the Fmoc/*t*Bu, protecting group strategy, and converted to D-Bac8c(Leu2,5)-N_3_ **18** by diazotransfer reaction with imidalole-1-sulfonyl azide hydrochloride and cleavage from the resin. Finally, a click reaction between **22** and **18** was performed in solution leading to the formation of the target cephalotin- D-Bac8c(Leu2,5) **15**. Conjugate **15** is considered a prodrug since the carbamate moiety of the conjugate acts as a cleavable linker when the β-lactam ring is hydrolyzed in the presence of β-lactamases, delivering the free peptide.

However, even if the final conjugate **15** could be potentially used in systemic therapies preventing toxicity issues, it was found to have a slightly lower MIC than the parent-free peptide against *E. coli* and MRSA, probably due to a lower uptake.

**Scheme 5:**
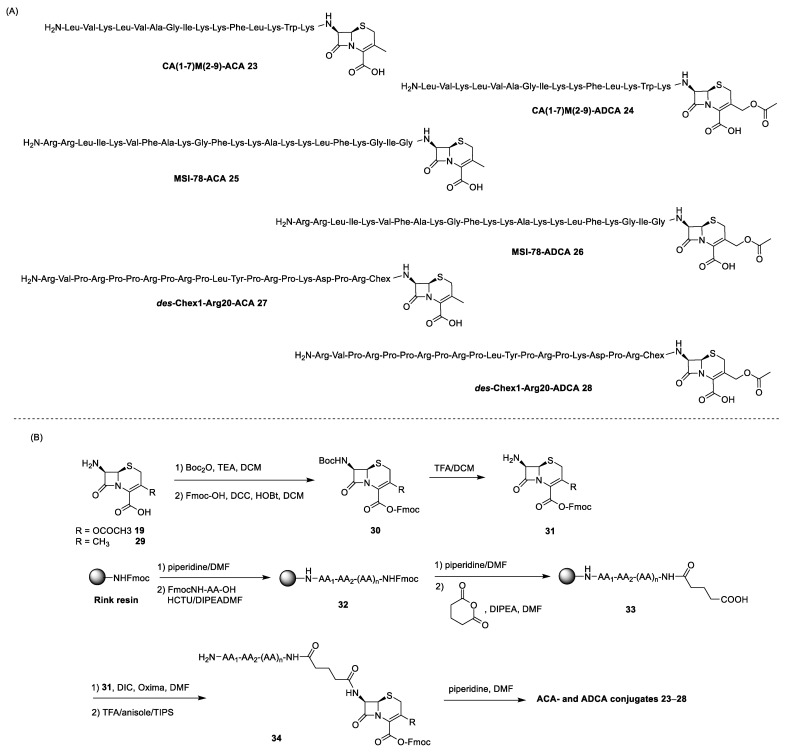
Structure of ACA- and ADCA-conjugates **23**–**28** (**A**) and their synthesis (**B**).

A second exploited point of conjugation on β-lactam antibiotics is the free amino function on the β-lactam ring. Accordingly, Wade et al. investigated the possibility of linking the N-terminus of three cationic AMPs, namely MSI-78, CA(1–7)M(2–9)NH_2_ and des-Chex1-Arg20, to the amino function of 7-ACA **19** and cephalosporins precursor 7-aminodesacetoxycephalosporanic acid (7-ADCA) **29** directly to the solid phase through the glutaric acid linker, producing 6 AMP-β-lactam conjugates **23**–**28** (Scheme 5A) [105]. Accordingly, by protecting the amino function of 7ACA and 7-ADCA as NH-Boc carbamate and the carboxylic acid as Fmoc-ester, they obtained intermediates **30** that were selectively Boc-deprotected, generating derivatives **31** that can be readily used in SPS (Scheme 5B). The AMPs were grown on Rink resin and after Fmoc-deprotection of the last amino acid of **32**, they were coupled with glutaric anhydride, leading to the formation of resin-bound peptides **33**, which were coupled with **31**, cleaved from the resin producing **34**, and finally deprotected at the carboxylic function of the β-lactams in a solution affording conjugates **23**–**28**. The activity of these conjugates was measured against different nosocomial pathogens and only in one case did the conjugate MSI-78-ACA-**25** and MSI-78-ADCA **26** reveal a synergistic effect against *A. baumannii* and *MDR A. baumannii 156*.

**Scheme 6:**
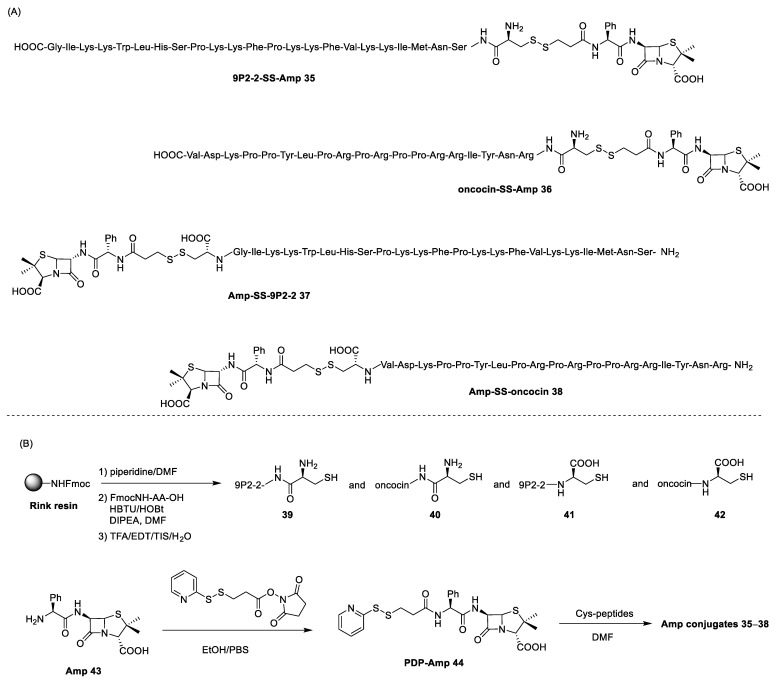
Structure of Amp-conjugates **35**–**38** (**A**) and their synthesis (**B**).

Another small library of 4 β-lactam antibiotic–AMP conjugates was synthetized exploiting the free amino group on the β-lactam ring and a stimuli-responsive disulfide linker [106]. The rationale behind the design of such conjugates is to exploit the ability of AMPs to cross the inner and outer bacterial cell membranes of Gram-negative bacteria to help the β-lactam antibiotic reach its targets after the cleavage of the disulfide linker in the periplasm and cytosol [107]. Accordingly, ampicillin (Amp), herein used as a model β-lactam antibiotic, was tethered at either the N- and C-terminus of two AMPs having different characteristics, namely membrane-disrupting magainin analog 2P2-2 that was developed by the same group [108] and proline-rich oncocin, which is able to cross the inner and outer membranes without membrane lysis [109], producing the four conjugates **35**–**38** represented in Scheme 6A. Amp **43** was functionalized with 3-(2-pyridyldithio)propionic acid *N*-succinimidyl ester (PDPS) to obtain intermediate **44**, which was coupled in solution with the two AMPs that were previously synthetized through SPPS and tagged with the required Cys, either at the N- and C-terminus, yielding the target Amp-AMP conjugates **35**–**38**, having a cleavable disulfide linker (Scheme 6B). The MIC values of the four conjugates, along with undecorated AMPs, physical mixtures of AMP + Amp, and conjugates built with a non-cleavable thioether linker, were evaluated against Gram-negative bacteria *E. coli* BW 25113 and *A. baumannii* ATCC 19606 and Gram-positive *Staphylococcus epidermidis* ATCC 12228. Interestingly, derivative Amp-SS.9P2-2 **37** showed significantly increased activity against Amp-resistant *A. baumannii* and no cytotoxicity against HEK cells, whereas the oncocin-conjugates did not show enhanced antimicrobial activity, probably due to a lower membrane permeability induced by the introduction of the Cys tag on the peptide sequence. It is worth noting that conjugate **37** is the first β-lactam-AMP conjugate that showed remarkably increased activity against ampicillin-resistant Gram-negative bacteria, highlighting the efficiency of an approach based on tethering-suitable antibiotics with ad hoc AMPs through a cleavable linker. 

### 4.3. Aminoglycoside–AMP Conjugates

**Figure 6:**
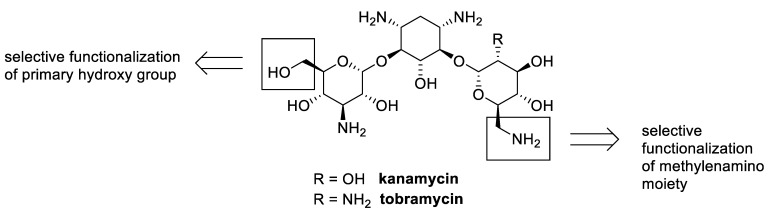
Kanamycin and tobramycin selective functionalization.

Aminoglycosides (AGs) have a broad spectrum of activity against pathogenic bacteria, which has led to their extensive use and occasional misuse over the past seventy years, establishing them as a valuable class of antibiotics. However, their clinical effectiveness is hampered by the toxic side effects associated with their use, in particular nephrotoxicity and ototoxicity, and by the evolution of different mechanisms of resistance developed by bacteria, encompassing the decrease in AG uptake and the emergence of aminoglycoside-modifying enzymes (AMEs) [52,54]. Much effort has been devoted, and still is, to the chemical modification of natural aminoglycosides to accommodate a resurgence of these antibiotics. Similar to glycopeptide antibiotics, the chemical functionalization of aminoglycosides may initially appear challenging due to the presence of numerous identical functional groups, such as hydroxyl and amino groups, which exhibit similar reactivity. However, aminoglycosides contain primary alcohols and/or amino groups attached to primary carbons, which can be targeted for selective functionalization due to their lower steric hindrance (Figure 6). 

**Scheme 7:**
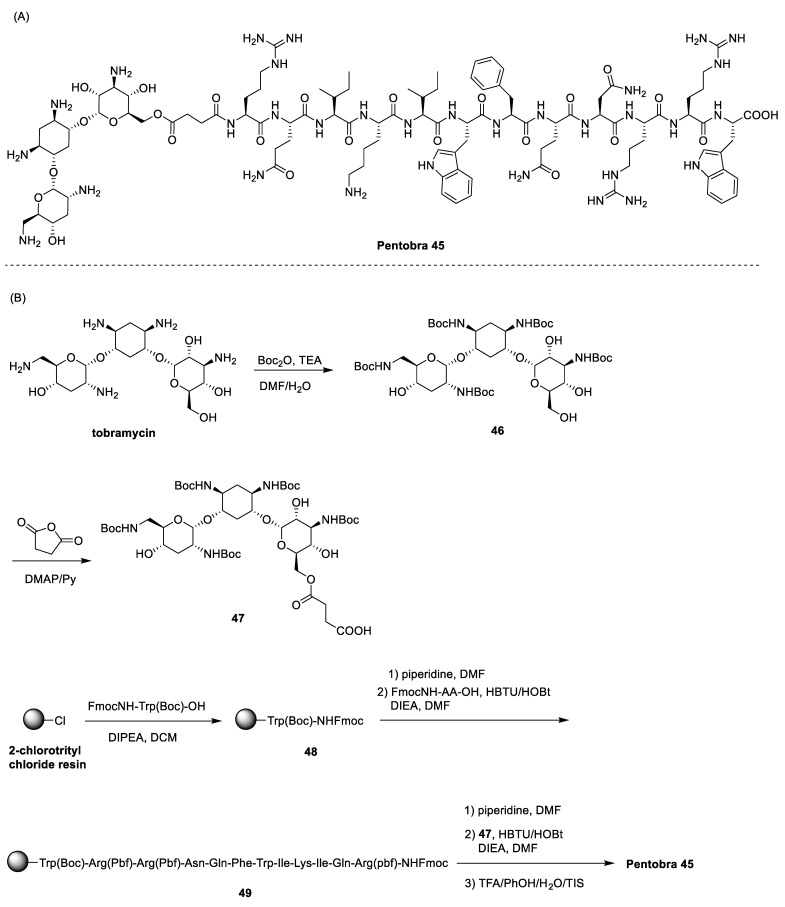
(**A**) Structure of tobramycin-AMP conjugate Pentobra **45**. (**B**) Synthesis of Pentobra **45**.

The first aminoglycoside-AMP conjugate that appeared in the literature, i.e., Pentobra **45** (Scheme 7A) was designed to target persister bacterial cells and to combat the anaerobic bacterium *Propionibacterium Acnes (P. acnes),* which is difficult to treat since charged antibiotics are not able to penetrate into the largely lipophilic sebaceous membrane [110]. To increase the bacterial permeability of tobramycin without missing its ribosomal activity, Pentobra **45** was designed by linking tobramycin to a short 12mer AMP with the ability to selectively permeate bacterial membranes through a succinyl linker [111,112]. Accordingly, after Boc-protection of the amino groups of tobramycin, the primary hydroxy group of **46** was selectively functionalized with succinic anhydride, leading to the formation of intermediate **47**, which was coupled in SP to the N-terminus of the AMP, produced after cleavage from the resin Pentobra **45** (Scheme 7B). The conjugate showed high activity against *E. coli* and *S. aureus* persister cells and a wide range of *P. acnes* due to the synergic effects, namely membrane activity and inhibition of protein synthesis [111], along with no adverse effect and anti-inflammatory activity [112].

**Scheme 8:**
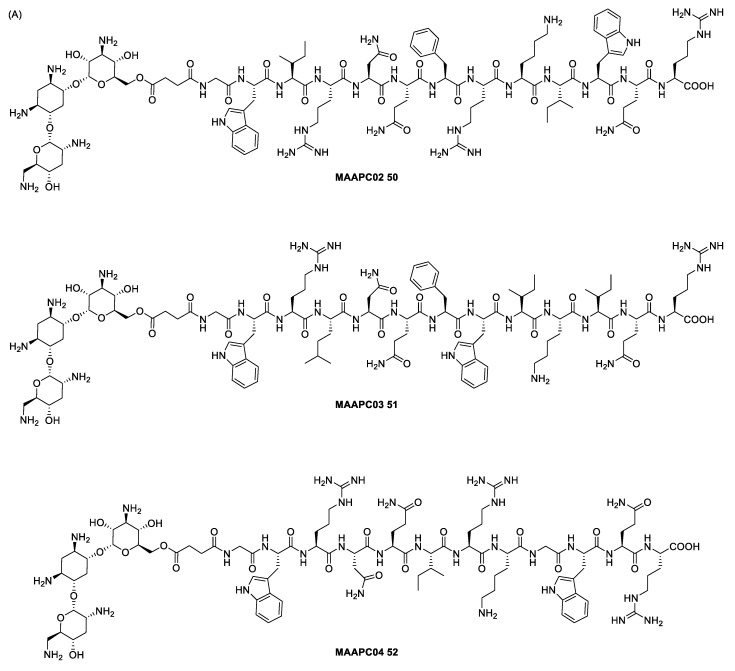

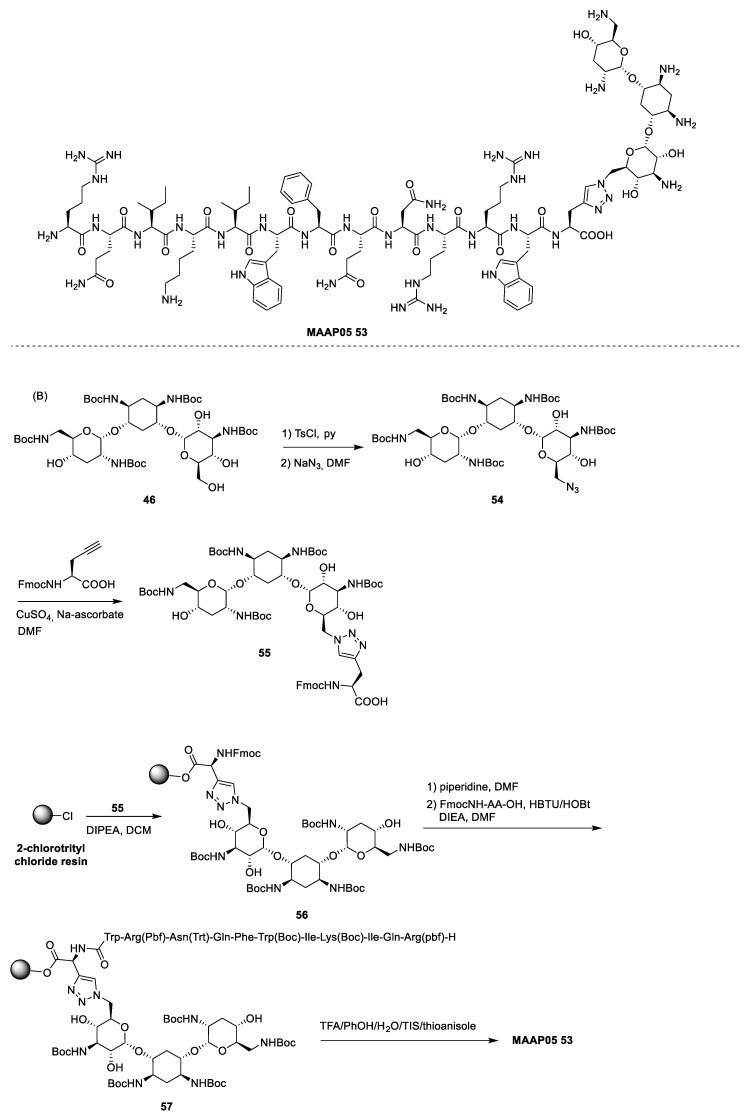
(**A**) Structure of tobramycin conjugates **50**–**53**. (**B**) Synthesis of MAAP05 53.

In the attempt to increase the potentiality of Pentobra **45** in terms of accumulation in bacteria by increasing membrane permeability and limiting the action of the efflux systems activated by the bacterial species, the same group synthetized a collection of four new kanamycin-AMP conjugates, MAAP02-05 **50**–**53**, where the peptide transporter sequence is modified according to sequence principles based on quantum mechanical models for membrane-permeating peptides (Scheme 8A) [113]. Probably due to the low yield obtained in the functionalization of tobramycin for the synthesis of Pentobra **45**, a new synthetic pathway was employed. Accordingly, the primary hydroxy group of Boc-protected tobramycin **46** was selectively transformed in tosylate upon treatment with tosyl chloride in pyridine and substituted by an azide, leading to the formation of intermediate **54** which was clicked with Fmoc-NH-protected propargyl-alanine-affording conjugate **55** (Scheme 8B). The free carboxylic acid was used to anchor **55** to 2-cholotrytyl chloride resin (CTC) where the AMP peptide was grown through Fmoc-strategy, providing after cleavage the synthesis of two 13-mer and two 12-mer tobramycin-AMP conjugates, referred to as MAAPCs **50**–**53**. The MAAPCs demonstrated good selectivity for bacterial cell membranes over mammalian cell membranes and did not cause significant hemolysis of human red blood cells. They also exhibit superior antibacterial activity against actively growing Gram-negative *E. coli* compared to Gram-positive *S. aureus*. Among them, MAAPC05 **53**, along with Pentobra **45**, exhibits the highest inner membrane permeability, which correlates well with antimicrobial activity against persisters, showing much better activity than tobramycin alone.

**Scheme 9:**
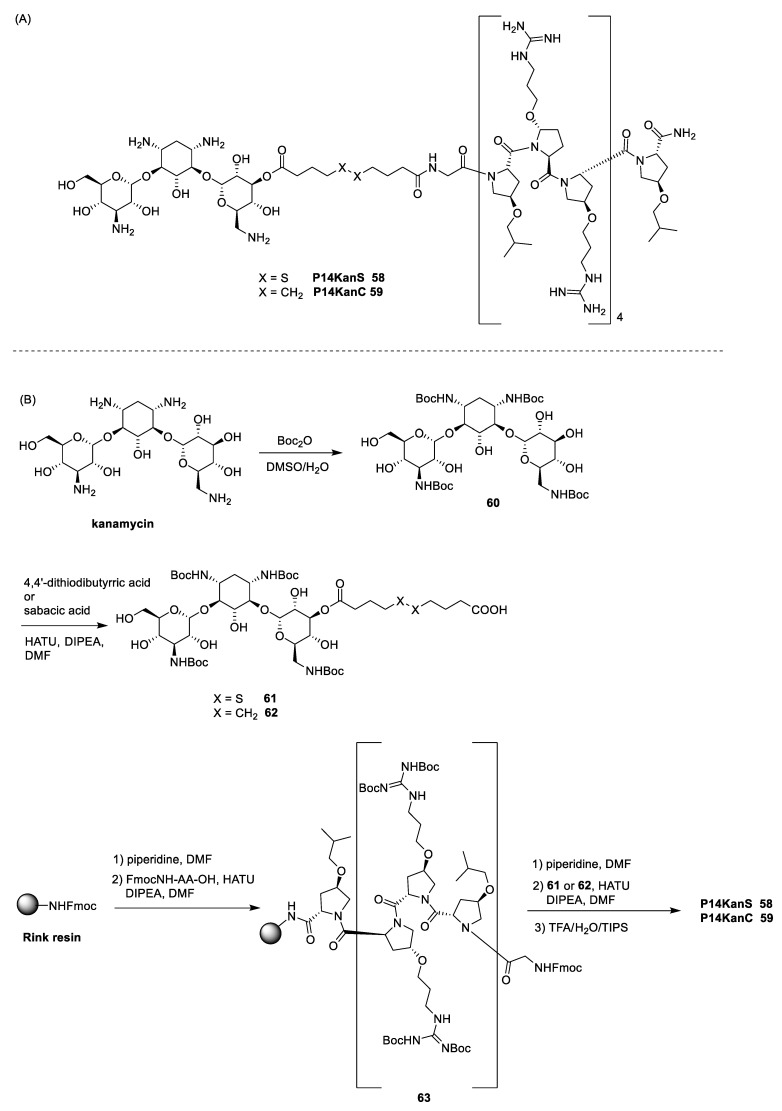
(**A**) Structure of P14KanS **58** and P14KanC **59**. (**B**) Synthesis of P14KanS **58** and P14KanC **59**.

With the same rationale, namely to increase the ability of aminoglycosides to cross bacterial cell membranes, aminoglycoside-AMP conjugates were designed to fight bacterial pathogens encompassing MRSA, *Salmonella*, *Mycobacterium*, and *Brucella*, which are internalized within mammalian cell macrophages [114,115,116]. Since aminoglycosides, along with other antibiotics, are characterized by insufficient membrane permeability within macrophages and suffer drug efflux, kanamycin was tethered to a modified proline-rich cell-penetrating peptide with intrinsic, nonmembrane lytic antimicrobial activity targeting intracellular pathogenic bacteria [117], through both a cleavable disulfide linker or non-cleavable alkyl linker, generating P14kanS **58** and P14kanC **59**, respectively, (Scheme 9A) [118]. Boc-protected kanamycin **60** was reacted with 4,4′-dithiobutyric acid or sebacic acid, giving rise to the formation of mixtures of isomers from which compounds **61** and **62** were isolated and fully characterized by NMR spectroscopy. The obtained intermediates **61** and **62** were coupled in SP to the N-terminus of the proline-rich AMP, and generated after cleavage from the resin the target conjugates P14kanS **58** and P14kanC **59** (Scheme 9B). Very interestingly, P14kanS **58** was more potent than P14kanC **59**, P14LRR AMP, and the non-covalent mixture of kanamycin and P14LRR against different Gram-negative and Gram-positive bacteria, encompassing intracellular pathogens. Since the conjugates did not lyse membranes, as demonstrated by monitoring the β-galactosidase release from *E. coli* after the addition of the conjugates, these results demonstrated the synergistic effect of the antibiotics, which can operate when the disulfide bond is cleaved in the reductive environment inside the cell, and kanamycin is released. Moreover, in successive work, P14kanS **58** has proved to have potent antimicrobial activity against ESKAPE pathogens, along with anti-inflammatory activity and a great ability to treat biofilms [119].

**Scheme 10:**
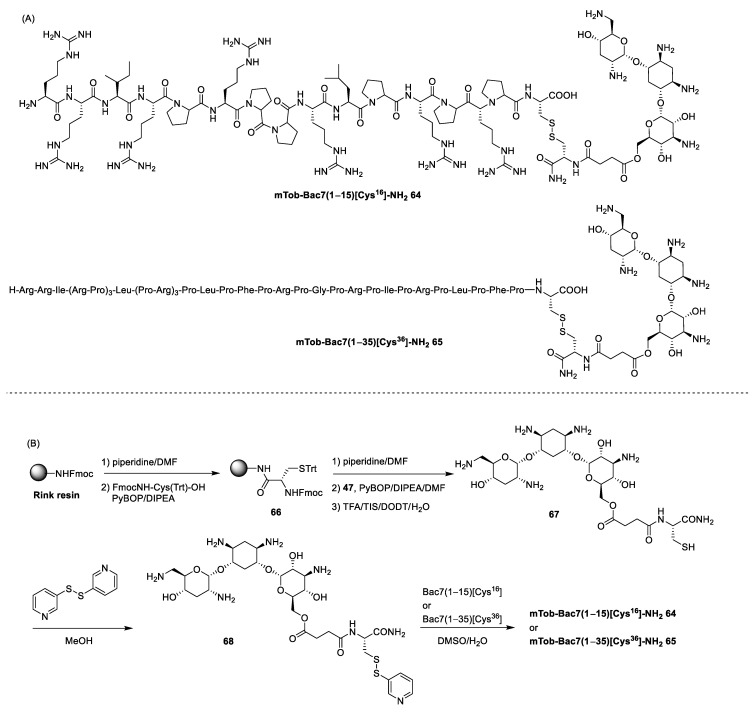
(**A**) Structure of tobramycin-Bac7 conjugates **64**, **65**, and (**B**) their synthesis.

Another proline-rich antimicrobial peptide (PrAMP) is Bac7, and in particular, the segments Bac7(1–16) and Bac7(1–35), the 16-mer and 35-mer N-terminal segments that showed comparable antimicrobial activity to the parent full peptide [120,121]. These functional fragments are referred to as bacteria-penetrating peptides (BPPs) since they cross the bacterial inner membrane via the SbmA transporter without permeabilizing the membrane at active concentrations to eventually interact with the target ribosome and inhibit protein synthesis. To target bacterial ribosomes with two distinct synergistic mechanisms, Bac7(1–16) and Bac7(1–35) fragments were tethered to tobramycin with a cleavable disulfide linker that would release the active components in the intracellular reductive environment (Scheme 10A) [122]. Resin-bound Cys **66**, which was obtained by anchoring FmocNH-Cys(Tr)-OH to the Rink resin, was Fmoc-deprotected and coupled with succinyl Boc-trobamycin **47**, obtained as described in Scheme 7B, leading to the formation of tobramycin-Cys conjugate **67** after cleavage from the resin (Scheme 10B). Conjugate **67** was reacted with 2,2′-dithiopyridine to yield **68**, which was submitted to conjugation with Bac7(1–15)[Cys^16^]NH_2_ and Bac7(1–35)[Cys^36^]NH_2,_ producing the final hybrid antibiotics mTob-Bac7(1–15)[Cys^16^]NH_2_ **64** and mTob-Bac7(1–35)[Cys^36^]NH_2_ **65**, respectively. The resulting conjugates showed activity against strains to which tobramycin and the Bac7 segments were inefficient, such as clinically isolated Gram-negative bacteria strains *E. coli* and *P. aetuginosa,* and other Gram-negative species (*A. baumanii* and *S. enteridis*), proving that the conjugation strategy is rewarding even if the real mechanism of action is not yet clear.

**Figure 7:**
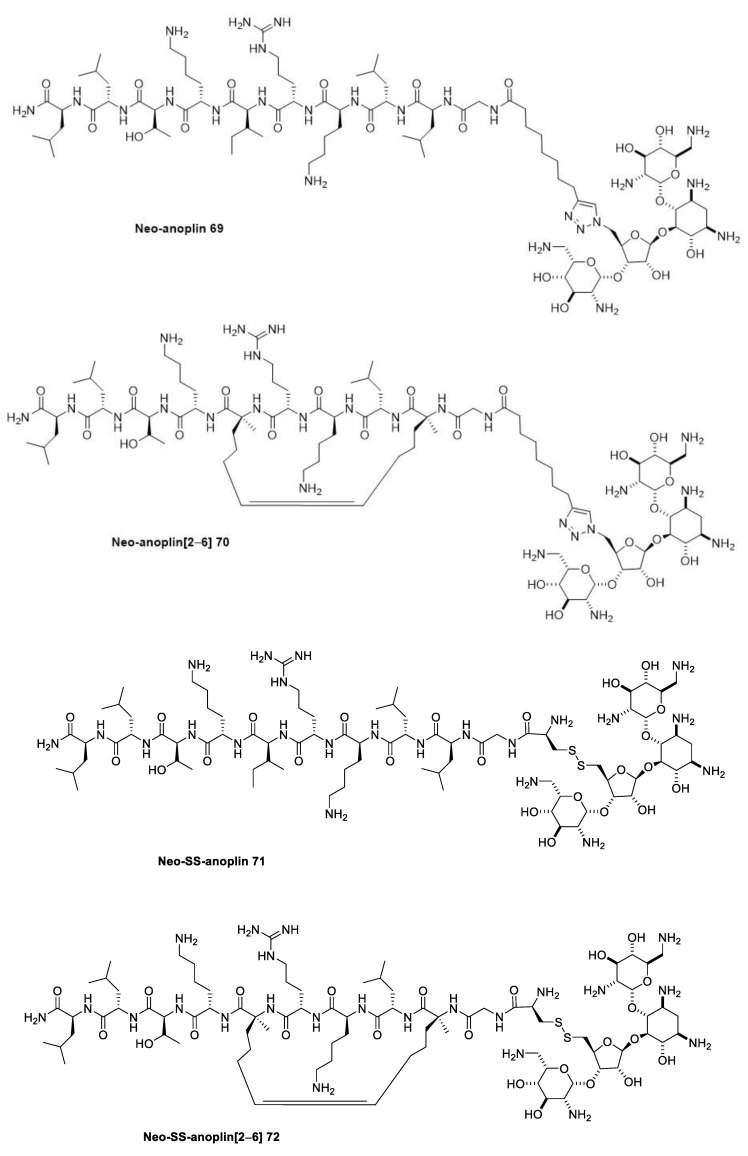

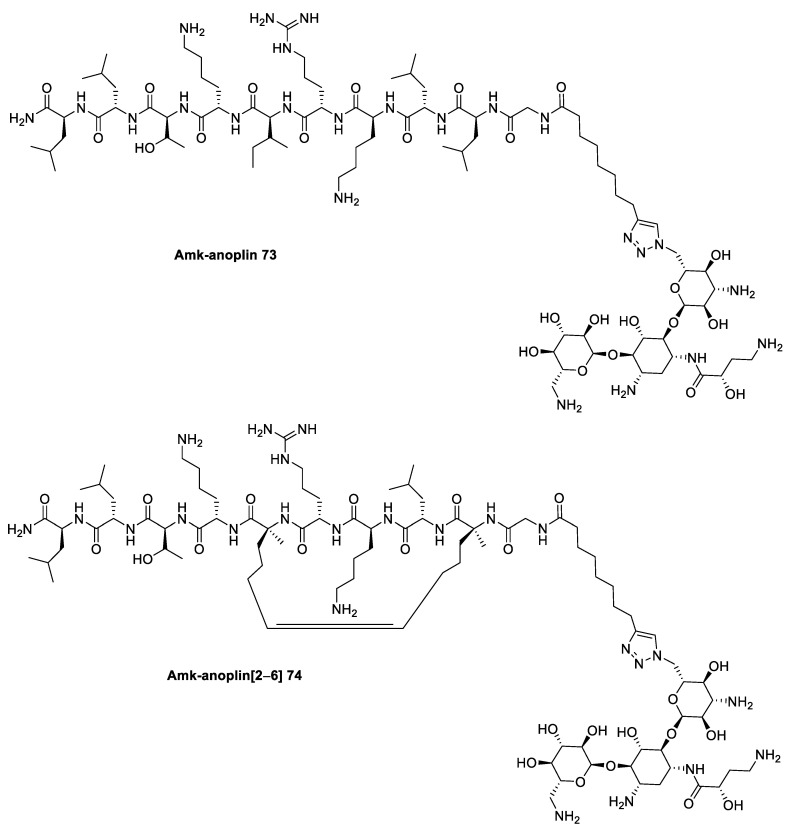
Structure of Neo- and Amk-anoplin conjugates **69**–**74**.

The antibacterial activity of PrAMPs depends also on the propensity of such peptides to assume more stable secondary conformations that have been shown to be very important to their ability to permeate and destabilize the bacterial cell membrane. A common strategy to stabilize the secondary structure of peptides, other than the introduction of prolines in the sequence, is peptide stapling [123], a technique that has been successfully exploited in the design of active stapled antimicrobial peptides (StAMPs) [124]. A peptide that has witnessed an improvement in terms of proteolytic stability and antibacterial activity, thanks to the stabilization of its helical structure upon hydrocarbon stapling, is anoplin [125]. Recently, anoplin and stapled anoplin have been tethered to amikacin and neomycin through both a non-cleavable triazole linker and a cleavable disulfide linker, generating a small library of two non-cleavable neomycin-anoplin conjugates, namely Neo-anoplin **69** and Neo-anoplin[2–6] **70**, two cleavable neomycin-anoplin conjugates, i.e., Neo-SS-anoplin **71** and Neo-SS-anoplin[2–6] **72**, and two non-cleavable amikacin-anoplin conjugates, i.e., Amk-anoplin **73** and Amk-anoplin[2–6] **74**, whose structures are reported in Figure 7 [126].

**Scheme 11:**
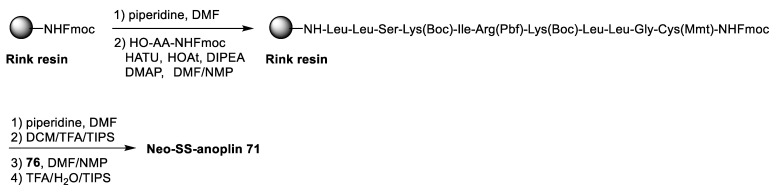
Synthesis of Neo-anoplin **69**, Neo-anoplin[2–6] **70**, and Neo-SS-anoplin **71**.

For the synthesis of these conjugates, the presence of only one primary hydroxy group on both the aminoglycosides neomycin and amikacin was exploited. As an example, neomycin was first Boc-protected at the amino functions, then reacted with bulky triisopropylsulfonyl chloride (TIPS-Cl) to selectively transform the primary hydroxy group in sulfate which was transformed into the corresponding azide **75** by nucleophilic substitution (Scheme 11). Azide **75** can be either clicked with anoplin or anoplin[2–6], functionalized at the N-terminus with dec-9-ynoic acid for the synthesis of the non-cleavable derivatives **69** and **70**, respectively, or reacted with thiourea followed by 2-mercaptopryridined to afford intermediate **76**, which were reacted in solution to anoplin or anoplin[2–6], functionalized at the N-terminus with Cys to afford cleavable Neo-SS-anoplin **71** and Neo-SS-anoplin[2–6] **72**, respectively. In this case, the conjugates obtained, regardless of the nature of the linker and the structure of the AMP, were only slightly more active, or as active as the corresponding components, showing no synergistic effect.

**Scheme 12:**
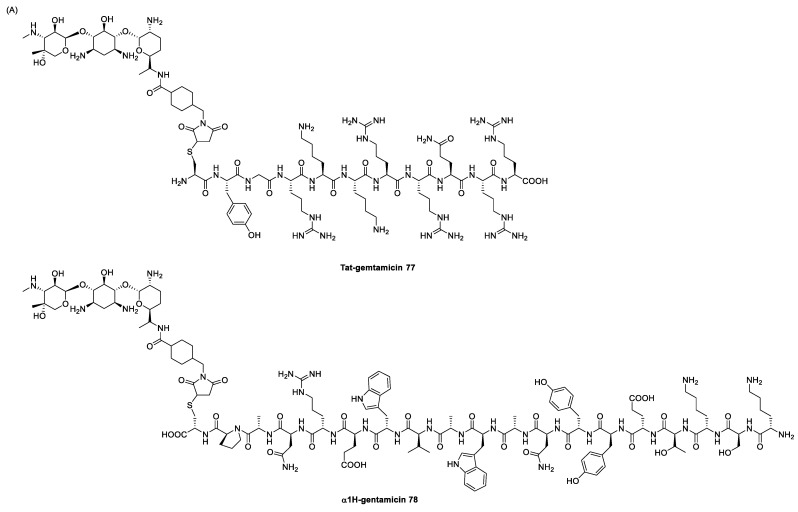

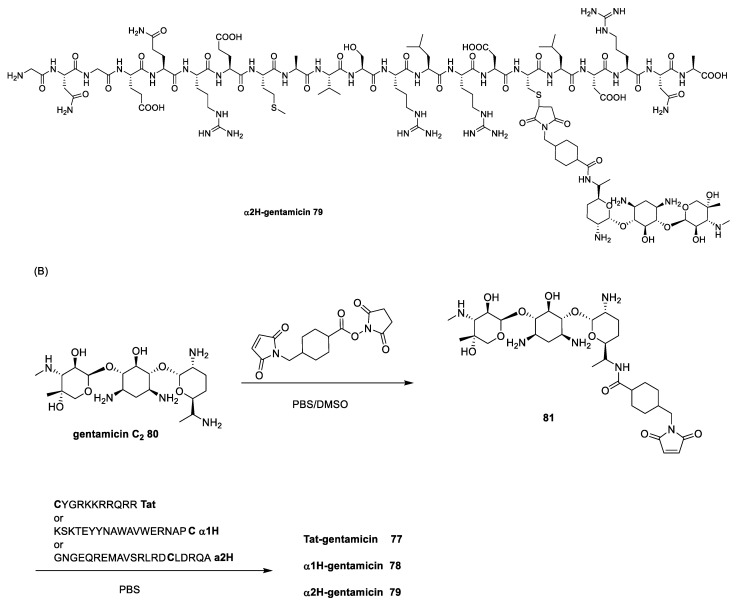
(**A**) Structure of CPP-gentamicin conjugates **77**–**79**, and (**B**) their synthesis.

The improvement of the uptake of aminoglycosides to make them able to fight intracellular bacterial infections has also been explored in combination with CPPs. In particular, two peptides, α1H and α2H, which are two α-helices responsible for the penetration ability of the bacterial effector protein YopM into eukaryotic cell [127], have been tethered to gentamycin through a non-cleavable linker producing two conjugates, namely α1H-gentamycin **77** and α2H-gentamicin **78**, along with a third conjugate synthetized by anchoring gentamycin to the well-known polyarginine Tat peptide, i.e., Tat-gentamycin **79** (Scheme 12A) [128]. Accordingly, by reacting gentamycin **80** with cross-linker succinimidyl-4-(N-maleimidomethyl)cyclohexane-1-carboxylate (SMCC), the intermediate **81** was obtained as the main product (Scheme 12B). Conjugate **81** was then clicked through thiol-maleimide chemistry to Cys-modified Tat, α1H, and α2H peptides, leading to the formation of the final conjugates **77**–**79**, respectively.

Both α1H, α2H, and Tat peptides were able to promote cellular internalization of gentamycin since the corresponding conjugates **77**–**79** were active against multiple intramolecular Gram-negative pathogenic bacteria, such as *E. coli* K1, *Salmonella enterica,* and *Shigella flexneri*.

### 4.4. Miscellanous

Apart from β-lactams, vancomycin, and aminoglycosides, other classes of antibiotics have also been used to build antibiotic–AMP conjugates.

**Scheme 13:**
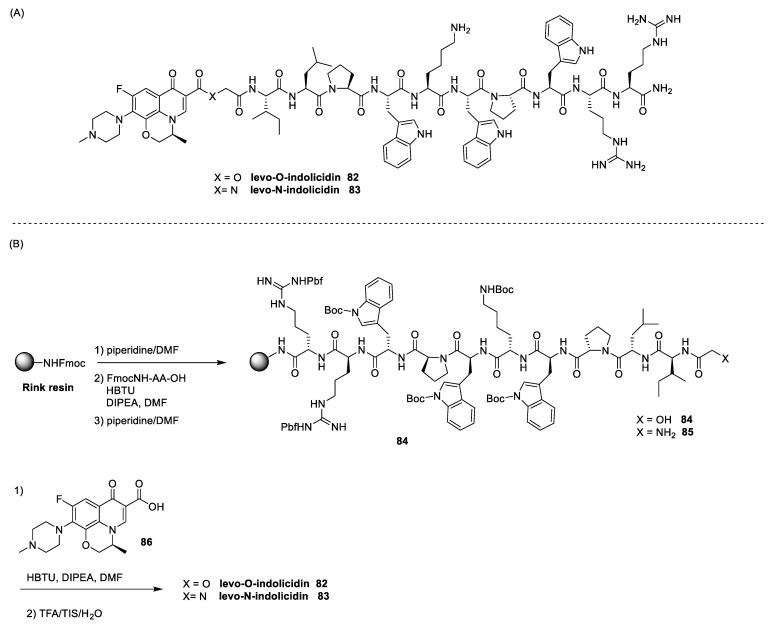
(**A**) Structure levofloxacin-indolicidin conjugates **82** and **83** and (**B**) their synthesis.

Inspired by the observation that when fluoroquinolones are administered with AMPs, the resulting cocktail shows a synergistic effect, broadening the antibacterial spectrum of the antibiotics along with a decreasing therapeutic dose that would result in lower adverse reactions [120,129], Toh et al. reasoned that a similar result could be obtained by linking levofloxacin to indolicin—an AMP with a broad spectrum of activity against Gram-negative and Gram-positive bacteria [130]—through a labile ester linkage or a more stable amide linker. The conjugation produced two AMP-levofloxacin conjugates, namely the prodrug levo-O-indolicidin **82** and the corresponding amide derivative levo-N-indolicidin **83**, whose ability to cross the outer membrane of bacteria could be higher than that of levofloxacin due to the present of the highly lipophobic peptide (Scheme 13A) [131]. The two conjugates **82** and **83** were synthetized through SPPS by reacting the free carboxylic acid of levofloxacin **86** with the N-terminus of the peptide tagged with glycolic acid or Gly **84** and **85**, respectively (Scheme 13B). While the physical mixture of indolicin and levofloxacin was slightly more active compared to both antibiotics, in particular against *B. subtilis* ATCC 6633, the conjugates did not show the same effect.

**Figure 8:**
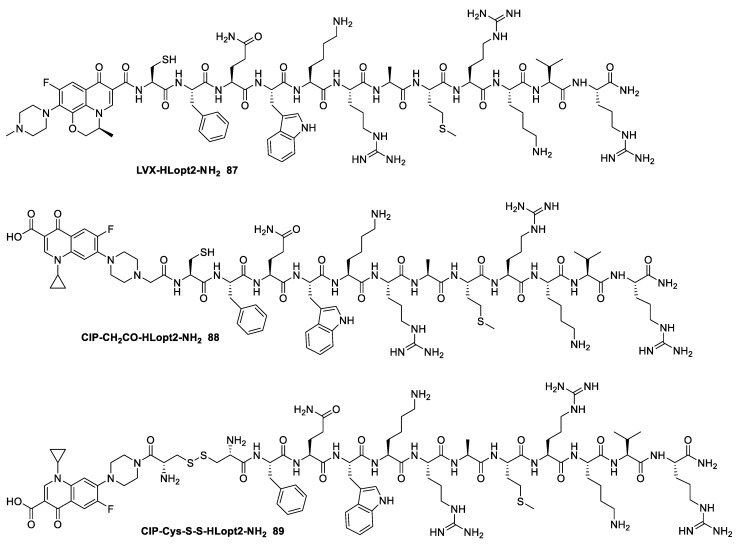
Structure of HLopt2 conjugates **87**–**89**.

Levofloxacin, along with another fluoroquinolone ciprofloxacin, was also tethered to a different AMP, namely HLopt2, which is an antimicrobial analog of HLP-2, a segment of Lactoferrin with potent antimicrobial activity against both Gram-negative and Gram-positive bacteria [132]. The three conjugates were designed and synthetized to increase the permeability of the fluroquinolone antibiotics thanks to the ability of HLopt2 to destroy the bacterial cell through pore formation mechanisms (Figure 8) [133]. All the conjugates were synthetized through SPPS, LVX-HLopt2-NH_2_ **87** by anchoring the carboxylic acid of levofloxacin to the N-terminus of HLopt2, CIP-CH2CO-HLopt2-NH_2_ **88** by coupling the secondary amine of ciprofloxacin with the N-terminus of HLopt2 previously functionalized with bromoacetic acid, while CIP-Cys-SS-HLopt2-NH_2_ **89**, the only conjugate with a stimuli-responsive linker, by formation of a disulfide bond between Cys-modified ciprofloxacin and Cys residue, was linked to the N-terminus of HLopt2 [134]. Interestingly, all the conjugates showed increased activity along with low toxicity to mammalian cells and very low hemolytic activity, CIP-Cys-SS-HLopt2-NH_2_ **89** being the most active against *S. aures* due to the reducing environment that trigged the disulfide bridge cleavage with the corresponding release of the two antibiotics.

**Scheme 14:**
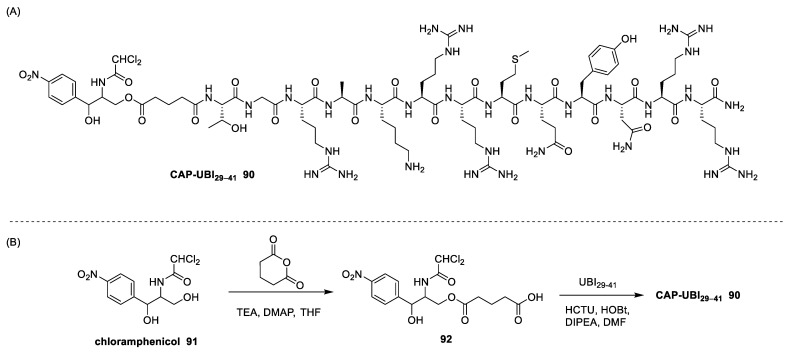
(**A**) Structure of CAP-UBI_29–41_ **90** and (**B**) its synthesis.

Other than to increase the permeability of small-molecule antibiotics, the conjugation strategy with AMP could be exploited to overcome the non-specificity of potent broad-spectrum antibiotics which suffer severe toxic side effects [135]. For instance, chloramphenicol (CAP) is one of the most effective broad-spectrum antimicrobial agents whose clinical use was hampered by its high risk of bone marrow toxicity [136]. CAP, being lipid-soluble, diffuses through the bacterial cell membrane and reversibly binds to the L16 protein of the 50S subunit of bacterial ribosomes. This binding prevents the transfer of amino acids to growing peptide chains, likely by suppressing peptidyl transferase activity, thereby inhibiting peptide bond formation and subsequent protein synthesis. To overcome its non-specificity, chloramphenicol was tethered to UBI_29–41,_ which is a cationic AMP highly investigated for its capacity to bind bacteria with high affinity [137], through a non-cleavable glutaric linker, leading to the formation of CAP-UBI_29–41_ **90** (Scheme 14A) [138].

CAP **91** was reacted with glutaric anhydride, yielding a mixture of products due to the indiscernible reactivity of the two hydroxy groups, from which intermediate **92** was isolated at around 50% yield. After that, **92** was coupled in solution with commercially available UBI29-41 yielding, after HPLC purification, CAP-UBI_29–41_ conjugate **90**. Gratifyingly, in vitro studies demonstrated that CAP-UBI_29–41_ **90** has enhanced antibacterial effects on *S. aureus* and *E. coli*., also showing significantly reduced toxicity to normal cells compared to CAP. Most importantly, this result was also obtained in bacteria-bearing mouse models, indicating that UBI29-41 is an ideal targeting ligand for constructing antibacterial agents for bacteria-targeting therapy.

**Scheme 15:**
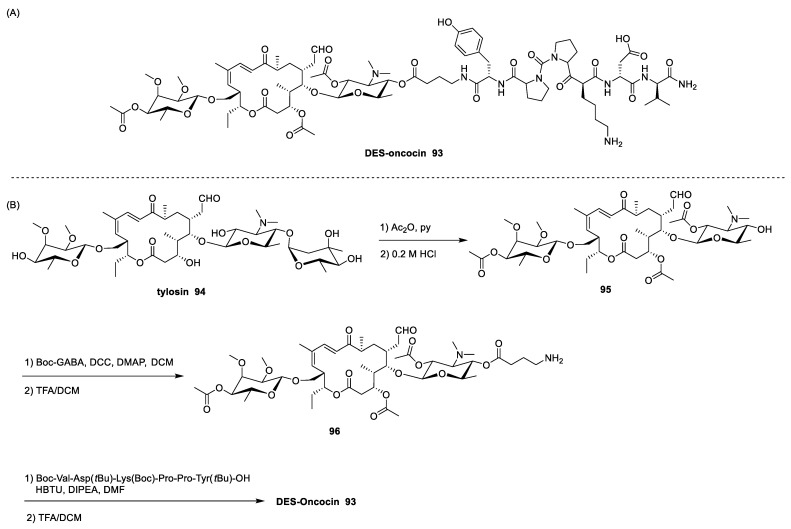
(**A**) Structure of DES-oncocin **93** and (**B**) its synthesis.

Differently from CAP, selectivity is not a big issue for the use of macrolides, since their mechanism of action depends on their affinity to the so-called “macrolide-binding site” which allows them to selectively inhibit translation in bacteria. However, this class of antibiotics suffers antibiotic resistance due to the ability of bacteria to modify the target-binding site [58]. To fight antibacterial resistance, a huge body of work has been devoted to chemically modifying the different scaffolds of macrolides. For instance, the modification of the 4′- and 4″-hydroxyl groups of the mycaminose moiety of desmycosin (DES) leads to analogs able to fight antibacterial resistance [139]. With the same aim, DES was conjugated to fragments of oncocin, an AMP whose activity depends on the interaction with a binding site that overlaps with the binding site of macrolides [109,140]. DES-oncocin **93** (Scheme 15A) was synthetized starting from tylosin antibiotic **94**, which was acetylated and hydrolyzed under acidic conditions, producing DES derivative **95 and** having the hydroxyl group in the 4′ position unprotected, thus ready to be selectively functionalized (Scheme 15B) [141]. Accordingly, **95** was coupled with the Boc-γ-aminobutyric acid (GABA) linker, affording, after deprotection, intermediate **96**, which was coupled with three different oncocin-fragments, the longer being Boc-Val-Asp(tBu)-Lys(Boc)-Pro-Pro-Tyr(tBu)-OH previously prepared through SPPS, yielding the target conjugate **93** after deprotection of all the side chains.

The resulting conjugates showed activity against some macrolide-resistant bacteria strains by binding to the *E. coli* 70S ribosome, thus inhibiting bacterial protein synthesis and suppressing bacterial growth.

## 5. Conclusions

Despite their widespread use and success in treating bacterial infections, conventional antibiotics face several significant challenges. One of the primary issues is the rapid development of bacterial resistance, which can render these drugs ineffective over time. The discovery and development of new antibiotics have slowed down significantly due to high costs, lengthy development times, and stringent regulatory requirements, making it difficult to keep pace with emerging resistant strains. AMPs are an alternative to small-molecule antibiotics, offering several promising advantages in combating bacterial infections and antimicrobial resistance. One of their primary benefits is their broad-spectrum activity, allowing them to target a wide range of pathogens, including bacteria, fungi, and viruses. AMPs typically act rapidly with a mode of action that is less specific than traditional antibiotics, reducing the likelihood of resistance development. Moreover, they can disrupt biofilms, which are protective layers formed by bacterial communities that conventional antibiotics often cannot penetrate. However, AMPs also suffer from some drawbacks, such as susceptibility to proteolytic degradation by enzymes in the human body, cytotoxicity towards human cells at higher concentrations, and poor pharmacokinetic properties, necessitating frequent dosing or alternative administration routes, which can be less convenient for patients.

Combination therapy with old antibiotics and AMPs represents a promising frontier in the fight against AMR, overcoming most of the drawbacks associated with the use of single components. This innovative therapy combines the potent, broad-spectrum activity of AMPs with the targeted efficacy of traditional antibiotics, resulting in enhanced antimicrobial effectiveness. The choice between antibiotic–AMP conjugates and coadministration depends on various factors, including the type of infection, the specific pathogens involved, patient characteristics, and available resources. Coadministration provides flexibility and simplicity but requires careful management to avoid issues with drug interactions and resistance. Conjugates offer the potential for highly targeted and effective treatment with lower resistance development, but they come with challenges related to complexity and cost. By leveraging the membrane-active properties of AMPs, antibiotic–AMP conjugates facilitate improved antibiotic penetration and intracellular concentration, while also ensuring more specific targeting of bacterial cells over host cells. This dual-action approach not only increases the potency of the treatment but also allows for lower dosages, reducing potential side effects and minimizing the risk of developing resistance.

Although no antibiotic–AMP conjugates have successfully reached the market thus far, as research continues, with ongoing advancements in technology, innovations in peptide synthesis, delivery methods, and conjugation technologies, alongside a deeper understanding of bacterial biology and the mechanisms by which AMPs and small-molecule antibiotics operate, antibiotic–AMP conjugates are emerging as a versatile and powerful strategy for addressing the global challenge of AMR.

Peptide synthesis technologies are evolving, allowing for the creation of more complex and stable AMPs with enhanced therapeutic properties. Advances in delivery methods are ensuring that these conjugates can be effectively transported to the target sites within the body, maximizing their efficacy while minimizing potential side effects. Additionally, new conjugation technologies are enabling the precise linkage of AMPs and antibiotics, enhancing their synergistic effects and overcoming bacterial defenses more effectively.

Our growing understanding of bacterial biology is crucial in this fight. Insights into bacterial resistance mechanisms, biofilm formation, and virulence factors are guiding the design of more effective conjugates. By targeting specific bacterial pathways and structures, these conjugates can disrupt the bacteria’s ability to survive and replicate, even in the presence of traditional antibiotics. Furthermore, the mechanisms of action of AMPs and antibiotics are being elucidated in greater detail. This knowledge is instrumental in creating conjugates that can bypass resistance mechanisms, such as efflux pumps and enzymatic degradation, which bacteria commonly use to neutralize antibiotics. By combining the membrane-disrupting properties of AMPs with the intracellular-targeting capabilities of antibiotics, these conjugates can deliver a one-two punch that bacteria find difficult to counteract. Additionally, the versatility of antibiotic–AMP conjugates lying in their potential to be tailored for specific infections and bacterial strains can lead to more effective treatments with fewer side effects, as therapies can be designed to target only the pathogenic bacteria without harming the beneficial microbiota. This precision medicine approach not only enhances patient outcomes but also reduces the likelihood of resistance development.

Finally, economic and regulatory incentives must play a critical role in the development and deployment of antibiotic–AMP conjugates. Governments and health organizations are recognizing the urgent need to combat AMR and are providing funding, tax incentives, and streamlined regulatory pathways to accelerate the development of new antimicrobial agents. These incentives are crucial for encouraging pharmaceutical companies to invest in this high-risk, high-reward area of research and development.

In conclusion, the integration of cutting-edge technologies, a deepening understanding of bacterial biology, and supportive economic and regulatory frameworks are paving the way for antibiotic–AMP conjugates to become a cornerstone in the fight against AMR. These conjugates hold promise for revitalizing the efficacy of existing antibiotics and introducing new, potent antimicrobial therapies to safeguard public health for future generations.

## Data Availability

No new data were created or analyzed in this study.
